# Changes in Medicare Accountable Care Organization Spending, Utilization, and Quality Performance 2 Years Into the COVID-19 Pandemic

**DOI:** 10.1001/jamanetworkopen.2023.5237

**Published:** 2023-03-29

**Authors:** Brandon W. Yan, Maya Shashoua, Jose F. Figueroa

**Affiliations:** 1 School of Medicine, University of California, San Francisco; 2 Department of Health Policy and Management, Harvard T.H. Chan School of Public Health, Boston, Massachusetts; 3 Department of Medicine, Brigham & Women’s Hospital and Harvard Medical School, Boston, Massachusetts

## Abstract

This cohort study uses data from the Medicare Shared Savings Program to assess changes in spending, utilization, and quality performance from before the COVID-19 pandemic (2019) to year 2 of the pandemic (2021).

## Introduction

The Medicare Shared Savings Program (MSSP), Medicare’s largest accountable care organization (ACO) program, cares for 11 million Medicare beneficiaries.^1^ Despite the COVID-19 pandemic, the program returned a record $1.9 billion in net savings to Medicare in 2020, a feat made possible by an unprecedented decline in spending and utilization for non–COVID-19 care.^2^ While net savings from the MSSP decreased to $1.7 billion in 2021, 81% of the 475 reconciled ACOs still achieved gross savings compared with cost benchmarks.^3^ This cohort study examined whether spending and utilization recovered to pre–COVID-19 levels in 2021 and, importantly, assessed how quality of care has evolved.

## Methods

This study was considered exempt from review because it used data from the 2019 to 2021 MSSP public use files. We analyzed the ACO program’s per capita spending overall and across inpatient, postacute, and outpatient care, with COVID-19–related spending excluded per MSSP financial accountability rules (eAppendix in Supplement 1). We also analyzed changes in utilization, quality performance, and patient experience measures. Adjusted mean differences were derived from linear regression models comparing ACO performance in 2019 vs 2020 and 2021 (eAppendix in Supplement 1). Because 2019 included new ACO contracts with redesigned participation options that were implemented in July 2019, analyses were weighted for half-year or full-year contract lengths. The significance threshold was 2-tailed *P* = .05. The study followed the Strengthening the Reporting of Observational Studies in Epidemiology (STROBE) reporting guideline.

## Results

In 2021, mean total non–COVID-19 spending per capita recovered statistically to 2019 levels after an 8.5% decline in 2020 (*P* < .001) (Table 1). However, mean acute inpatient spending in 2021 remained 12.4% lower than 2019 levels (*P* < .001), while outpatient spending increased to 3.8% higher than 2019 levels (*P* < .001). Postacute spending remained statistically flat. Mean utilization remained lower than pre–COVID-19 levels across acute inpatient discharges, postacute discharges, and emergency department (ED) visits, but primary care use returned to baseline. Mean quality performance in 2021 decreased to 5.1 points lower than 2019 levels (*P* < .001). Nonetheless, the MSSP had improved screening rates for depression, falls, and tobacco use as well as increased influenza vaccination. Mean blood pressure control remained worse in 2021 than in 2019, while diabetes control recovered to 2019 levels (Table 2). Patient experience scores decreased across nearly all measures from 2019 to 2021.

**Table 1.** Mean ACO Performance in the MSSP From 2019 to 2021 Across Spending and Utilization

Measure	2019 (n = 680)	2020 (n = 513)	2021 (n = 475)	2019-2020			2019-2021		
				Adjusted change from 2019 to 2020 (95% CI)	Percent change	*P* value	Adjusted change from 2019 to 2021 (95% CI)	Percent change	*P* value
Spending, $ per capita									
Total	11 496.1	10 536.5	11 290.0	−976.0 (−1131.1 to −820.9)	−8.5	<.001	−98.2 (−257.3 to 60.9)	−0.9	.23
Acute inpatient care^a^	3098.8	2637.7	2648.7	−441.0 (−494.8 to −387.3)	−14.2	<.001	−384.4 (−442.4 to −326.5)	−12.4	<.001
Postacute care^b^	2027.0	1973.8	1939.8	−45.2 (−124.7 to 34.3)	−2.2	.27	12.0 (−63.0 to 87.1)	−0.6	.75
Outpatient care^c^	6284.7	5810.7	6587.5	−526.4 (−604.7 to −448.2)	−8.4	<.001	237.8 (150.1 to 325.5)	3.8	<.001
Utilization, per 1000 person-years									
Acute inpatient discharges^d^	293.6	252.8	247.4	−37.0 (−40.7 to −33.2)	−12.6	<.001	−36.9 (−40.8 to −32.9)	−12.6	<.001
Postacute care discharges^e^	76.4	67.0	64.9	−8.6 (−12.1 to −5.0)	−11.3	<.001	−7.3 (−10.1 to −4.5)	−9.6	<.001
ED visits	926.4	772.2	801.7	−134.8 (−145.7 to −123.9)	−36.1	<.001	−82.1 (−93.6 to −70.6)	−8.9	<.001
Primary care services	10 970.7	10 234.1	10 974.9	−843.6 (−1059.2 to −628.0)	−7.7	<.001	−65.4 (−296.6 to 165.9)	−0.6	.58

Abbreviations: ACO, accountable care organization; ED, emergency department; MSSP, Medicare Shared Savings Program.

^a^ Acute inpatient spending is the sum of short-term acute care hospital spending, inpatient psychiatric hospital spending, and other inpatient spending.

^b^ Postacute care spending is the sum of long-term care hospital spending, hospice spending, skilled nursing facility or skilled nursing unit spending, inpatient rehabilitation facility spending, and home health spending.

^c^ Outpatient spending is the sum of outpatient spending and physician and/or supplier spending.

^d^ Acute inpatient discharge utilization is the sum of short-term hospital utilization and psychiatric hospital or psychiatric unit utilization.

^e^ Postacute care discharge utilization is the sum of long-term hospital utilization, rehabilitation hospital or rehabilitation unit utilization, and skilled nursing facility utilization.

**Table 2.** Mean ACO Performance in the MSSP From 2019 to 2021 Across Quality Measures

Measure	2019 (n = 680)	2020 (n = 513)	2021 (n = 475)	2019-2020			2019-2021		
				Adjusted change from 2019 to 2020 (95% CI)	Percent change	*P* value	Adjusted change from 2019 to 2021 (95% CI)	Percent change	*P* value
Overall quality score^a^	94.7	97.8	90.0	3.0 (2.7 to 3.3)	3.2	<.001	−5.1 (−5.8 to −4.4)	−5.4	<.001
Specific quality measures, %									
Poorly controlled diabetes^b^	13.7	14.7	12.8	1.4 (0.8 to 2.1)	10.2	<.001	0 (−0.8 to 0.9)	0	.96
Controlled high blood pressure^c^	75.3	72.9	74.8	−2.9 (−3.7 to −2.0)	−3.9	<.001	−1.4 (−2.2 to −0.5)	−1.9	.003
Depression screening and follow-up plan	71.1	71.5	74.0	0.4 (−1.5 to 2.3)	0.6	.67	2.9 (0.9 to 4.8)	4.1	.004
Screening for future fall risk	84.6	85.0	87.0	−0.1 (−1.6 to 1.4)	−0.1	.91	1.6 (0.1 to 3.1)	1.9	.04
Influenza immunization	75.1	76.0	80.5	0.2 (−1.0 to 1.5)	0.3	.73	4.2 (3.0 to 5.3)	5.6	<.001
Tobacco use screening and cessation intervention	78.3	81.7	81.0	3.2 (1.4 to 5.0)	4.1	.001	2.4 (0.4 to 4.3)	3.1	.02
Colorectal cancer screening	71.2	72.6	73.6	0.5 (−0.7 to 1.7)	0.7	.41	0.6 (−0.6 to 1.8)	0.8	.33
Breast cancer screening	74.2	74.0	75.1	−0.9 (−2.0 to 0.1)	−1.2	.09	−0.8 (−1.9 to 0.3)	−1.1	.14
Statin therapy for prevention and treatment of CVD	82.4	83.4	84.2	0.9 (0 to 1.8)	1.1	.05	1.7 (0.8 to 2.5)	2.1	<.001
Depression remission at 12 mo	13.7	14.0	15.5	0.2 (−1.3 to 1.7)	1.5	.82	1.6 (0 to 3.2)	11.7	.05
Patient experience scores^a^^,^^d^									
Receipt of timely care, appointments, and information	86.0	NA	84.7	NA	NA	NA	−1.3 (−1.7 to −0.8)	−1.5	<.001
Clinician communication	93.9	NA	93.5	NA	NA	NA	−0.3 (−0.6 to −0.1)	−0.3	.001
Clinician rating	92.4	NA	92.2	NA	NA	NA	−0.3 (−0.5 to −0.1)	−0.3	.003
Access to specialists	81.8	NA	78.8	NA	NA	NA	−2.9 (−3.3 to −2.5)	−3.5	<.001
Health promotion and education	60.3	NA	61.6	NA	NA	NA	1.1 (0.6 to 1.7)	2.2	<.001
Shared decision-making	62.6	NA	60.9	NA	NA	NA	−1.7 (−0.1 to −1.2)	−2.7	<.001
Health and functional status	73.6	NA	71.8	NA	NA	NA	−2.1 (−2.3 to −1.8)	−2.9	<.001
Care coordination	86.5	NA	85.7	NA	NA	NA	−0.9 (−1.2 to −0.6)	−1.0	<.001
Courteous and helpful office staff	92.5	NA	91.9	NA	NA	NA	−0.6 (−0.9 to −0.3)	−0.6	<.001
Stewardship of patient resources	26.3	NA	24.7	NA	NA	NA	−1.5 (−2.0 to −0.9)	−5.7	<.001

Abbreviations: ACO, accountable care organization; CVD, cardiovascular disease; MSSP, Medicare Shared Savings Program; NA, not available.

^a^ Score range, 0-100.

^b^ Poorly controlled diabetes was defined as hemoglobin A_1c_ level greater than 9%.

^c^ Controlled high blood pressure was defined as blood pressure lower than 140/90 mm Hg.

^d^ Patient experience measures were temporarily excluded in 2020 and reintroduced in 2021.

## Discussion

In Medicare’s largest ACO program, non–COVID-19 health care spending per capita in 2021 recovered to the pre–COVID-19 baseline after a steep decline in 2020 but with a relative shift from acute inpatient to outpatient spending. The $1.7 billion in net savings to Medicare compared with MSSP cost targets seem to come from sustained reductions in inpatient, postacute facility, and ED utilization into year 2 of the COVID-19 pandemic.^3^ Despite a decrease in postacute facility discharges, the lack of statistical reduction in postacute care spending is explained in part by a coinciding increase in hospice spending. Notably, initial concerns about a post–COVID-19 surge in admissions from delayed care have thus far not materialized in the MSSP.^4^

Quality performance decreased in 2021 vs 2019, reflecting a reduction in patient experience scores (reintroduced for 2021 after a 2020 suspension due to the COVID-19 pandemic). Notable declines occurred in scores for timely care, specialist access, and shared decision-making, which may reflect system challenges with managing the rebound in outpatient utilization amid workforce shortages and fatigued clinicians. Nevertheless, 99% of ACOs met the quality threshold for shared savings eligibility.^3^ In addition, the MSSP’s better-than-expected performance in sustaining breast and colon cancer screening rates during the pandemic suggests financial accountability could successfully facilitate the provision of preventive care.^3,5,6^ However, more progress is needed in chronic disease control. Study limitations include a lack of data on spending outside the MSSP to compare performance and a lack of COVID-19 spending data to assess true total spending. Moving forward, the exclusion of COVID-19–related care from financial accountability should be revisited as endemicity takes hold, utilization patterns shift, and high-value COVID-19 care becomes a reasonable objective.

## Notes

Supplement 1. **eAppendix.** Statistical Analysis and CMS Definition of COVID-19–Related Care
eReferences

Supplement 2.
Data Sharing Statement
