# Supplementary material for: Changes in Medicare Accountable Care Organization Spending, Utilization, and Quality Performance 2 Years Into the COVID-19 Pandemic
Source: JAMA Netw Open. 2023 Mar 29;6(3):e235237. doi: 10.1001/jamanetworkopen.2023.5237 (PMC10061234; doi:10.1001/jamanetworkopen.2023.5237)
Supplement: Supplement 1. — eAppendix. Statistical Analysis and CMS Definition of COVID-19–Related Care eReferences [file jamanetwopen-e235237-s001.pdf]

Supplementary Online Content

Yan BW, Shashoua M, Figueroa JF. Changes in Medicare accountable care organization spending, utilization, and quality performance 2 years into the COVID-19 pandemic. *JAMA Netw Open*. 2023;6(3):e235237. doi:10.1001/jamanetworkopen.2023.5237

eAppendix. Statistical Analysis and CMS Definition of COVID-19–Related Care

eReferences

This supplemental material has been provided by the authors to give readers additional information about their work.

© 2023 Yan BW et al. *JAMA Network Open*.

eAppendix. Statistical Analysis and CMS Definition of COVID-19–Related Care

Statistical Analysis:

We used linear regression to compare mean spending, utilization, and quality scores between 2019 and 2020 and 2019 and 2021. Outcome measures are provided in Tables 1 and 2 of the manuscript and further described in the Medicare Shared Savings Program (MSSP) Public Use File Data Dictionary.[1] In the model, we adjusted for year-to-year changes in case mix using the Centers for Medicare & Medicaid Services' (CMS) average Hierarchical Condition Categories (HCC) scores for each ACO as well as ACO beneficiary age, sex, and race composition. Because MSSP's new contracting structure "Pathways to Success" was introduced in July 2019, performance year 2019 had ACO contracts under both the legacy MSSP and Pathways to Success structures. To account for contracts with half-year lengths, the analysis was weighted such that half-year contracts in 2019 were given a weight of 0.5 and full-year contracts were given a weight of 1.0 (which is also the weight given to all ACOs in 2020 and 2021 where this special situation does not apply). Robust standard errors were applied. Statistical significance was defined at the p=0.05 level. Analyses were performed using STATA 15.1 (College Station, TX).

CMS Definition of COVID-19 Related Care:

In response to the COVID-19 pandemic and public health emergency, CMS removed all Medicare Parts A and B payment amounts for episodes of care for treatment of COVID-19 from MSSP's benchmarking and cost performance assessment. Identification of a COVID-19 related episode of care is triggered by inpatient service for treatment of COVID-19, with the duration of the episode of care starting in the month of admission, through all inpatient stay months, and terminating the month following the end of the inpatient stay. This change resulted in the exclusion of all payment amounts for COVID-19 episodes of care from determination of benchmark year and performance year expenditures, including calculations of shared losses and shared savings, for MSSP ACOs.[2]

© 2023 Yan BW et al. *JAMA Network Open*.

eReferences

1. Performance Year Financial and Quality Results PUF Data Dictionary. CMS.gov. Accessed January 31, 2023. https://data.cms.gov/sites/default/files/2021-08/Performance_Year_Financial_and_Quality_Results_PUF_Data_Dictionary.pdf

2. Medicare Shared Savings Program: CMS Flexibilities to Fight COVID-19. Published online August 18, 2022. Accessed January 28, 2023. https://www.cms.gov/files/document/medicare-shared-savings-program-cms-flexibilities-fight-covid-19.pdf

© 2023 Yan BW et al. *JAMA Network Open*.
