# Supplementary material for: Changes in Medicare Accountable Care Organization Spending, Utilization, and Quality Performance 2 Years Into the COVID-19 Pandemic
Source: JAMA Netw Open. 2023 Mar 29;6(3):e235237. doi: 10.1001/jamanetworkopen.2023.5237 (PMC10061234; doi:10.1001/jamanetworkopen.2023.5237)
Supplement: Supplement 2. — Data Sharing Statement [file jamanetwopen-e235237-s002.pdf]

Data Sharing Statement

Data
Data available: Yes
Data types: Other (please specify)
Additional Information: The data underlying the results presented in the study are available from the Medicare Shared Savings Program Public Use File from CMS.
How to access data: https://data.cms.gov/medicare-shared-savings-program/performance-year-financial-and-quality-results
When available: With publication

Supporting Documents
Document types: None

Additional Information
Who can access the data: Data is publicly available for download.
Types of analyses: Data is publicly available for download.
Mechanisms of data availability: Data is publicly available for download.
